# Evaluation of Influencing Factors on the Maximum Climbing Specific Holding Time: An Inferential Statistics and Machine Learning Approach

**DOI:** 10.3390/jfmk7040095

**Published:** 2022-10-27

**Authors:** Carlo Dindorf, Eva Bartaguiz, Jonas Dully, Max Sprenger, Anna Merk, Stephan Becker, Michael Fröhlich, Oliver Ludwig

**Affiliations:** Department of Sports Science, Technische Universität Kaiserslautern, 67663 Kaiserslautern, Germany

**Keywords:** climbing, exhaustion, fatigue, training, machine learning, sports, gender, training, data mining, artificial intelligence

## Abstract

Handgrip strength (HGS) appears to be an indicator of climbing performance. The transferability of HGS measurements obtained using a hand dynamometer and factors that influence the maximal climbing-specific holding time (CSHT) are largely unclear. Forty-eight healthy subjects (27 female, 21 male; age: 22.46 ± 3.17 years; height: 172.76 ± 8.91 cm; weight: 69.07 ± 12.41 kg; body fat: 20.05% ± 7.95%) underwent a maximal pull-up test prior to the experiment and completed a self-assessment using a Likert scale questionnaire. HGS was measured using a hand dynamometer, whereas CSHT was measured using a fingerboard. Multiple linear regressions showed that weight, maximal number of pull-ups, HGS normalized by subject weight, and length of the middle finger had a significant effect on the maximal CSHT (non-dominant hand: R^2^_corr_ = 0.63; dominant hand: R^2^_corr_ = 0.55). Deeper exploration using a machine learning model including all available data showed a predictive performance with R^2^ = 0.51 and identified another relevant parameter for the regression model. These results call into question the use of hand dynamometers and highlight the performance-related importance of body weight in climbing practice. The results provide initial indications that finger length may be used as a sub-factor in talent scouting.

## 1. Introduction

Handgrip strength (HGS) appears to be a crucial factor in climbing success [1]. Although HGS has a major impact on climbing performance, it has also been shown to play the biggest role in bouldering, followed by lead climbing, then speed climbing [2]. Nevertheless, Ref. [3] criticizes the transferability of HGS, which is usually measured using a hand dynamometer and represents static and isometric force production, which is not quite similar to climbing-specific determinants, e.g., the maximal climbing-specific holding time (CSHT) [4]. In the field of general HGS research, measurements are commonly performed using hand dynamometers, and factors such as finger length [5], body weight [6] and constitution [7,8,9], hand volume [10], as well as age and gender [6,11] have been identified as possible determining factors. This could be an indication that these also determine the maximal climbing-specific holding time; however, empirical verification is still pending as only a few studies have investigated factors affecting CSHT [12].

Knowledge about factors influencing CSHT could be of relevance because these factors could have implications for better training methods or performance assessments. Therefore, in addition to classic inferential statistical approaches, machine learning modeling could further show the predictive power of respective parameters and be beneficial in terms of identifying new relevant parameters (see, e.g., [13,14]). To the best of our knowledge, there are currently only a few studies that have used ML methods in the context of sports climbing (e.g., in the classification and generation of routes for MoonBoard climbing [15]). Therefore, the actual potential of this methodology in the context of climbing seems to be largely unexamined.

Therefore, in this study, we analyzed whether there was a relation between different parameters (i.e., gender, weight, finger length, HGS, body fat, and maximal number of pull-ups) and maximal CSHT; we then tried to find a model that could predict the CSHT as accurately as possible using machine learning algorithms.

## 2. Materials and Methods

### 2.1. Subjects and Data Acquisition

Initially, data was collected from 52 subjects. For four subjects, the test was prematurely terminated because of their higher risk of injury as they were not able to hold their upper body in a stable position when hanging on the fingerboard. The data of these individuals were excluded from further analysis. Furthermore, illness and recent injuries in the upper extremities were exclusion criteria. For the final analysis, data from 48 healthy subjects were used (sex: 27 women, 21 men; age: 22.46 ± 3.17 years; height: 172.76 ± 8.91 cm; weight: 69.07 ± 12.41 kg; body fat: 20.05% ± 7.95%). Ten subjects had previous experience in sports climbing and trained one to three times a week without being active in sports climbing competitions. The other test participants were recreational athletes outside of climbing as well as people with little physical activity. The study was approved by the ethical committee of the university and met the criteria of the Declaration of Helsinki (World Medical Association, 2013). All participants signed informed consent forms, including permission to publish the results of the study. Subjects were asked not to perform intense physical activities for 48 h prior to the study and to come in a rested state.

### 2.2. General Measurement Procedure

Prior to the study, body composition was measured using a bioelectric impedance scale (InBody 770, InBody Europe, Eschborn, Germany). Height, upper and lower arm perimeter, and finger lengths were manually measured. Furthermore, the test subjects were asked for written information about their dominant (dH) and non-dominant (ndH) hand.

Additionally, using a 5-point Likert scale (0 = severely underdeveloped; 4 = well above average), the subjects were asked to what extent the following statements were true: (a) “My grip strength is heavily used in my everyday activities.” (b) “My grip strength is heavily used in my private (e.g., sporting) activities.” In addition, using an 11-point Likert scale (0 = severely underdeveloped; 10 = well above average), they were asked to provide subjective assessments of their CSHT.

Afterward, the maximum number of pull-ups was determined for each participant using a straight pull-up bar with radioulnar pronation of the palms. Correct execution criteria were chin at grip height at the upper position and full extension of the arms in the lower position. The grip width was set to shoulder width. Participants rested for at least 15 min before further measurements were taken after the pull-ups.

After the pull-ups, HGS was assessed using the Jamar hydraulic hand dynamometer (JLW Instruments, Chicago, IL, USA). Two measures of HGS were taken from each hand. We started with the dominant hand. Then, the non-dominant side was measured, before second measurements were carried out for each side. Following the method of [16], the maximum value achieved with either hand was used as the participant’s isometric HGS. For the determination of the reference values, measurements were carried out according to the instructions in [17]. Again, participants rested for at least 15 min before further measurements were taken after the HGS measurements.

**Figure 1:**
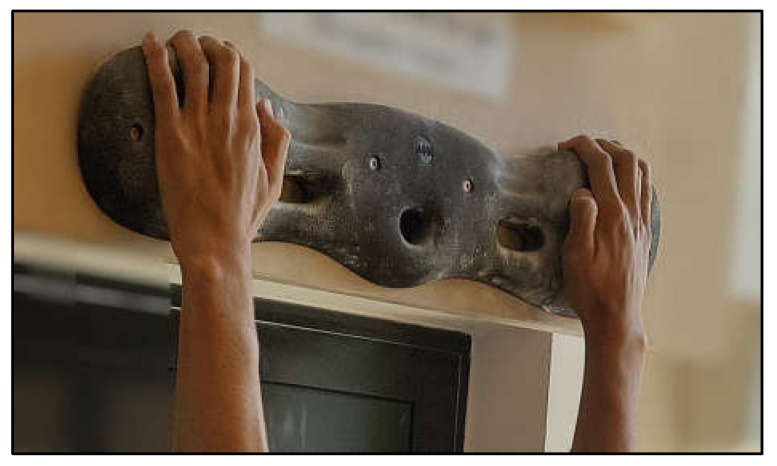
Hand positioning on the fingerboard.

The maximal CSHT was determined by measuring the maximal time without touching the ground on a four-centimeter-deep crimp with rounded edges and structuring (characteristics corresponded to typical climbing holds used in the gym) of a MOON fingerboard (Moon Climbing Limited, Sheffield, UK). Test participants maintained a dead hang position with straight arms with their feet lifted about 20 cm from the ground. The holds were cleaned before each run. Loose magnesium was directly applied to the fingers before carrying out the measurement, as this is performed in sports practice to improve friction [18]. The holding was performed without the use of the thumb, with three or four fingers, as habitually preferred by the subjects. An image of this is displayed in Figure 1. Directly after each termination (fall-off event), subjects were asked for subjective ratings of their perceived exertion using the OMNI scale [19].

### 2.3. Further Data Preparation and Calculations

Linear regression models for the modeling of CSHT were separately applied for the the dH and ndH variables to compare the results with each other. Backward elimination was applied to reduce the number of initial input variables. Dummy coding was performed for the categorical variable for sex (0 = woman, 1 = man). To consider possible multicollinearity, the following initial input variables were selected: sex, weight, body fat, maximal number of pull-ups, HGS normalized by the subject’s weight (dH and ndH), length of the middle finger (dH and ndH), and length of the little finger (dH and ndH). These were based on background knowledge derived from previous studies [5,6,7,11], as they seemed to have a high impact on HGS or CSHT. No auto-correlations were present according to Durbin–Watson statistics. Further necessary requirements were checked and could be assumed. Statistical analysis was performed using IBM SPSS (version 26, SPSS Inc., Chicago, IL, USA).

Modeling of the CSHT using machine learning was performed to evaluate the predictive performance of the measured variables, as well as further evaluate the selected variables in the final regression model. Therefore, elastic net regression was applied using the standard hyperparameters of Scikit-learn [20]. The elastic net model was especially appropriate when highly correlated features were present [21], as was the case with the available data. Explorative modeling was performed using all measured variables as input features (see Table 1).

The evaluation process was integrated into a leave-one-out cross- validation procedure for the evaluation of the model. The features were individually scaled through removal of the mean and scaling to unit variance based on each training set. Calculations were performed in Python (Python Software Foundation, Wilmington, DE, USA) and SPSS Statistics (version 25, SPSS Inc., Chicago, IL, USA).

## 3. Results

**Figure 2:**
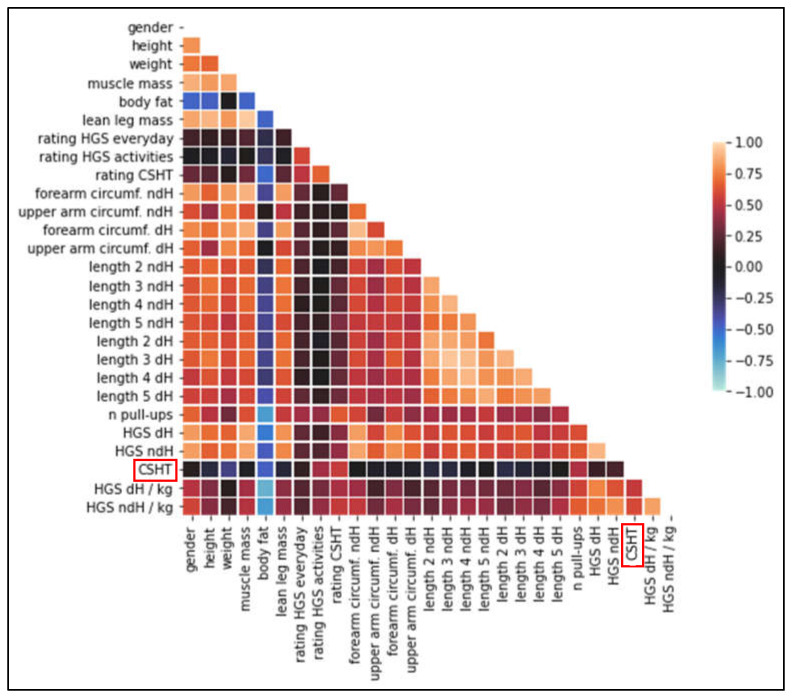
Descriptive correlation heat map of the measured variables. The color saturation represents the correlation strength (blue = negative correlation, yellow = positive correlation). The maximal climbing-specific holding time (CSHT) is highlighted with red boxes.

Subjects achieved slightly higher HGS scores for the dominant hand (41.69 ± 12.15 kg) compared with the non-dominant hand (38.99 ± 12.12 kg). On average, subjects rated their subjective estimated CSHT as 4.40 ± 1.73 on an 11-point Likert scale. Perceived exhaustion after treatment was rated moderately by subjects, at 5.06 ± 2.15 on the OMNI scale. The correlations of all measured variables are presented in Figure 2.

Using backward elimination separately for the dH and the ndH models, the same predictors were selected (weight, number of pull-ups, HGS, and length of middle finger). Multiple linear regression results showed that predictors demonstrated an effect on the climbing-specific holding time (dH: F(4,43) = 15.41, *p* < 0.001, *n* = 48, R^2^ = 0.59, R^2^_corr_ = 0.55; ndH: F(4,43) = 21.38, *p* < 0.001, *n* = 48, R^2^ = 0.67, R^2^_corr_ = 0.63). Detailed information regarding the predictors is presented in Table 2.

**Figure 3:**
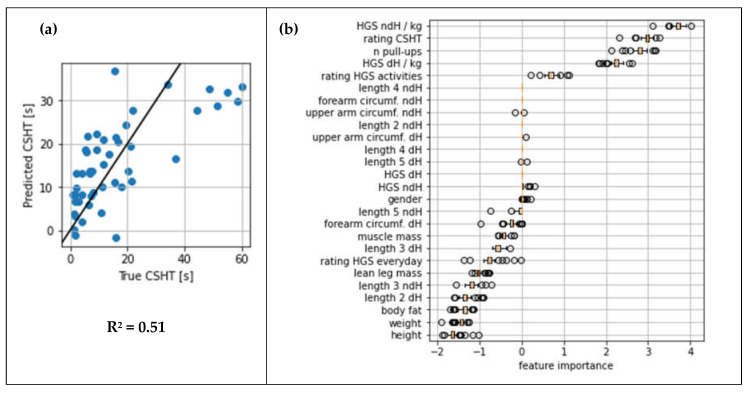
Results of the elastic net model. (**a**) True vs. predicted values for the CSHTS, as well as fit line for perfect fit. (**b**) Boxplots sorted according to the median coefficients (feature importance scores) of the features in the elastic net model during LOGO. Negative values represent an effect of high feature values reducing the CSHT; positive values represent an effect of high feature values increasing the CSHT. Please refer to Table 2 for feature labels.

Using machine learning for predictive modeling, an R^2^ score of 0.51 was achieved during cross-validation. Comparing true vs. predicted values (Figure 3a), we found that the model underestimated predicted values for subjects with a relatively high CSHT. The features in relation to the coefficients of the model are presented in Figure 3b. Most of the most important features regarding their coefficients matched with the variables in the final regression models. For example, sex was unanimously assigned no relevance by excluding it or receiving a score close to zero. The same applied, for example, to the parameter HGS ndH/kg, which showed a height effect for both approaches.

As we observed the importance of the subjective rating of the CSHT in Figure 3b, the additional inclusion of this parameter was evaluated in the regression analysis, as this aspect was previously not included. The fit slightly improved when including the subjective rating of CSHT (dH: F(5,42) = 14.44, *p* < 0.001, *n* = 48, R^2^ = 0.63, R^2^_corr_ = 0.59; ndH: F(5,42) = 19.32, *p* < 0.001, *n* = 48, R^2^ = 0.70, R^2^_corr_ = 0.66).

## 4. Discussion

The results of the current study showed that, of the predictors evaluated, weight, maximal number of pull-ups, HGS normalized by subject weight, and length of the middle finger had an especially significant effect on the maximal CSHT. The high agreement of the regression analysis between the dominant and non-dominant sides could be positively interpreted regarding the quality of the results. We found a slightly better model fit for the analysis of the ndH, which may indicate that aspects of the ndH are more limiting regarding the CSHT compared with aspects of the dH. This is probably related to the expected finding that subjects achieved higher HGS scores for the dominant hand compared with the non-dominant hand. It should be noted that a higher symmetry of HGS was found in climbers compared with non-climbing subjects [22].

The results also showed that HGS alone seems to be of little use in predicting CSHT, although some studies in the field of climbing refer to this parameter (e.g., [23]). Instead, HGS should be normalized to individual body weight, as shown by the high relevance of this parameter in both approaches. However, despite the inclusion of various other possibly relevant factors, as well as complex, non-linear modeling techniques derived from the field of machine learning, we could only obtain an R^2^_corr_ value of 0.63 for the non-dominant hand and an R^2^_corr_ of 0.55 for the dominant hand for the regression analysis and an R score of 0.51 for the predictive modeling using the elastic net model. According to [12], a high relative grip strength is essential for climbing routes of high difficulty, but high relative grip strength does not necessarily relate to higher climbing performance. Furthermore, comparing EMG measurements, the authors in [24] showed that HGS measurements using hand dynamometers showed a lack of specificity in mapping sports climbing characteristics. The authors in [3] explained the weak association between HGS and climbing-specific performance with the lack of specificity in most hand positions that are required during sports climbing (except pinch grip). Further research should therefore aim to apply feature engineering and develop optimal experimental designs to identify better predictive variables to improve model accuracy. Furthermore, the authors in [25] reported that grip strength depended on many more variables, such as bone density, VO_2max_, and the android-gynoid-fat-ratio, which we were not able to measure in this study, but which may also be related to CSHT. Additionally, Ref. [26] reported that climbing performance depends on flexibility, especially in the shoulder area. These variables were not measured in the current study and may be useful for improving model accuracy.

Finger length appeared to be a relevant factor for CSHT in the regression model results. As finger length is genetically determined, this could also be a crucial sub-factor when talent scouting. Therefore, the contributions of individual finger strength must be considered. The current findings, which showed that the length of the middle finger was a relevant parameter, are in line with a previous study [27] that explained that the middle finger produced the greatest force in hanging experiments. On the contrary, [28] found that ring finger strength was the most important individual finger strength measure for predicting climbing performance. In sports practice, it is sometimes suggested that the length of the little finger is crucial, as it affects how effectively the little finger can be used to hold crimps. In line with this notion, [27] reported that the little finger takes little more than 1/10 of the absolute force on climbing holds. Missing 1/10 of force can be significant in climbing, especially when climbing to exhaustion. However, the results of this study do not support the importance of the length of the little finger, as this parameter was not important in the final regression model. In the current study, some subjects were not able to keep their little finger on the holds of the fingerboard for a few seconds before termination, which could be explained by their progressive exhaustion during the dead hangs, leading to an inability to maintain the required hand position [3].

By identifying and adding the subjective rating of CSHT as a variable, the fit of the regression model could be further improved. Subjects seemed to be fairly good at assessing their abilities regarding the target parameter. Overall, the application of the ML model has shown great promise in expanding a background-knowledge-based regression model with data-driven knowledge concerning additional relevant parameters. Furthermore, we could confirm the suitability of the selected variables in the final regression model by the high level of agreement between the two approaches regarding the relevant parameters. This promising combination has also been demonstrated by other studies. In the context of cycling, for example, a combination of inferential statistical regression analysis and elastic net model has been used [29].

Both the regression model and the ML approach agree that sex had no relevant effect on the CSHT. This was surprising as sex differences were reported in HGS research [6,11]. Nevertheless, the results showed good fit with studies analyzing the forearm muscles during intermittent handgrip contractions, which is more specific to actual climbing loads, where they found no sex-specific differences in fatigability [30].

The authors in [26] showed that climbing performance was mainly related to training components, followed by anthropometric and flexibility components. Therefore, training status determines the CSHT to a significant degree. However, as found within the framework of the regression model, a person can also achieve a long holding time simply because of their low body weight. Accordingly, it cannot be generally said that people with longer CSHT are better-trained. To improve CSHT, the results suggest reducing body weight, as well as improving HGS and pull-up performance. However, climbing is characterized by a wide spectrum of conditional and coordinative abilities that are relevant to performance [31]. Therefore, isolated training of the parameters found appears to be insufficient for an optimal training strategy. According to [31], the best effect when undertaking conditioning training in climbing is obtained through a mixture of dynamic and static exercises in a semi-specific setting, combining hypertrophy, maximum strength, and endurance. In terms of more climbing-specific training, [32] states that interval bouldering works best as a form of sport-specific conditioning training, which may, in parallel, show positive effects on HGS and pull-up performance. In parallel with this approach, as shown by the results, attention should be paid to the athlete’s weight.

An underestimation of the predicted value for subjects with a relatively high CSHT was found for the elastic net model. This may be related to an underrepresentation of these subjects regarding their CSHT values in the training data. An extension of the training data through the further inclusion of related subjects could thus contribute to improving the model.

In the current study, we used CSHT as an indicator of climbing performance, as strong associations have been reported in the literature [4]. However, it is important to keep in mind that we can only make indirect statements about actual performance with this procedure. It is therefore unclear whether the analyzed factors predict actual climbing performance better than CSHT. Additionally, CSHT does not necessarily represent a realistic climbing situation, as the sport involves grip changes and short pauses between isometric contractions. Furthermore, the legs support a large amount of the climbers’ weight in a realistic climbing situation. In addition to the impact of anthropometric and fitness constitution, training age could also influence climbing performance. Research is therefore necessary to check if our results can be applied to more realistic climbing situations. In addition, other methods for the assessment of climbing-specific finger flexor strength, e.g., using a scale platform to measure the load that can be held by the test arm [33], may be helpful in gaining new insights.

## 5. Conclusions

The results of this study provide initial indications that finger length may play a role among many factors that map climbing talent in the context of talent scouting, but should not be seen in isolation from other potentially significant factors, such as technique or cognitive components. Furthermore, the results also confirm the performance-related importance of body weight, and thus, correspond to a general trend in competitive climbing, with extremely low body weights having been observed among these athletes and having been critically discussed in the context of anorexia athletica [34]. In the context of sports climbing training practice, the results indicate the potential relevance of regularly measuring the parameters of weight, maximum number of pull-ups, HGS normalized to body weight, and subjective assessment of CSHT to quantify training changes. Overall, the results support the potential usefulness of machine learning models as an exploratory tool in the context of sports science issues for enhancing inferential statistical analyses regarding the evaluation of predictive power, as well as the exploration and extension of existing knowledge.

## Figures and Tables

**Table 1:** Overview of the variables used as input features. dH = dominant hand; ndH = non-dominant hand.

Variable	Description
Gender Height Weight Muscle mass Body fat Lean leg mass	General anthropometric measurements
HGS dH HGS ndH	Hand grip strength (HGS) measured using hand dynamometer
HGS dH/kg HGS ndH/kg	HGS values normalized by subject weight
Rating HGS everyday Rating HGS activities Rating CSHT	Subjective ratings (see description in Section 2.2)
Forearm circumference ndH Forearm circumference dH Upper arm circumference ndH Upper arm circumference dH	Forearm and upper arm perimeters
Length 2 ndH Length 3 ndH Length 4 ndH Length 5 ndH Length 2 dH Length 3 dH Length 4 dH Length 5 dH	Individual finger length: 2 represents the index finger, 5 the little finger
*n* pull-ups	Maximal number of pull-ups
CSHT	Climbing-specific holding time, measured performing dead hang on finger board

**Table 2:** Regressors of the multivariate model to predict the maximal climbing-specific holding time (CSHT) for dominant (dH) and non-dominant (ndH) hands. In addition, the regression coefficients, B; standardized coefficients, β; standard error, SER (quality of the estimate); and *p*-values of the respective predictors are shown. HGS = hand grip strength.

	ndH R^2^_corr_ = 0.63				dH R^2^_corr_ = 0.55			
Predictors	B	β	SER	*p*	B	β	SER	*p*
Constant	60.94	-	18.82	0.002	66.84	-	21.45	0.003
Weight	−0.47	−0.35	0.15	0.003	−0.44	−0.33	0.17	0.013
Number of pull-ups	1.17	0.35	0.41	0.006	1.63	0.49	0.43	0.000
HGS/kg	33.66	0.55	7.31	0.000	22.74	0.39	7.33	0.003
Length 3 (middle finger length)	−7.38	−0.31	2.83	0.013	−7.15	−0.30	3.27	0.034

## Data Availability

The data are available if there is justified research interest.
